# Biocompatible and Implantable Optical Fibers and Waveguides for Biomedicine

**DOI:** 10.3390/ma11081283

**Published:** 2018-07-25

**Authors:** Roya Nazempour, Qianyi Zhang, Ruxing Fu, Xing Sheng

**Affiliations:** 1 Department of Electronic Engineering, Beijing National Research Center for Information science and Technology, Tsinghua University, Beijing 100084, China; rui-y16@mails.tsinghua.edu.cn; 2 School of Materials Science and Engineering, Tsinghua University, Beijing 100084, China; qy-zhang15@mails.tsinghua.edu.cn (Q.Z.); furx14@mails.tsinghua.edu.cn (R.F.)

**Keywords:** implantable devices, optical waveguides, optical fibers, biocompatible, biodegradable

## Abstract

Optical fibers and waveguides in general effectively control and modulate light propagation, and these tools have been extensively used in communication, lighting and sensing. Recently, they have received increasing attention in biomedical applications. By delivering light into deep tissue via these devices, novel applications including biological sensing, stimulation and therapy can be realized. Therefore, implantable fibers and waveguides in biocompatible formats with versatile functionalities are highly desirable. In this review, we provide an overview of recent progress in the exploration of advanced optical fibers and waveguides for biomedical applications. Specifically, we highlight novel materials design and fabrication strategies to form implantable fibers and waveguides. Furthermore, their applications in various biomedical fields such as light therapy, optogenetics, fluorescence sensing and imaging are discussed. We believe that these newly developed fiber and waveguide based devices play a crucial role in advanced optical biointerfaces.

## 1. Introduction

Optical fibers and waveguides are widely used in fiber-optic telecommunication, remote sensing and on-chip devices for a long time. With recent development of optical techniques towards medical applications, the interaction of light and living matter has always been favorable in a wide variety of medical purposes, such as laser surgery, phototherapy, biosensing and imaging [1,2,3,4,5,6,7,8,9,10]. However, the penetration depth of light in biological tissues at visible and near-infrared wavelengths is limited (<3 mm), due to absorption and scattering characteristics of tissues [11,12]. Implantable light sources, such as light emitting diodes and cell-based lasers [13,14,15], have been demonstrated for biomedical applications. Besides, injectable upconversion optoelectronic devices [16] or nanoparticles [17] have been developed to expand the optical penetration depth. However, these active implantable light sources still demand for further optimization to realize practical and clinical uses considering their sophisticated fabrication methods and biocompatibility issues. Alternatively, implantable fibers and waveguides provide an accessible way to deliver therapeutic or sensing light into deep tissues to overcome the penetration limit. Besides, optical fibers and waveguides are also serving the rapidly emerging photonic and optoelectronic implants to transmit optical signals and power [2,18]. In recent years, integrated optical fibers and waveguides have begun to emerge particularly in biomedical applications including optogenetics [19,20,21], fluorescence detection [22,23] and sensing [24,25]. Considering the impacts that these implanted devices might have on their host, the development of novel optical fibers and waveguides requires not only ideal optical properties, but also desirable biocompatibilities such as mechanical properties matching biological tissues, low cytotoxicity, minimally invasive injection, etc. In addition, optical fibers and waveguides made from biodegradable materials, which can physically disappear after use and eliminate the risk of further retraction, have attracted particular interests in the field.

Deriving from telecommunication, silica quartz based optical fibers have been used in biomedical field over decades [26,27]. The core-cladding structure in conventional silica fibers can be formed by the mature thermal drawing method and has the potential to deliver light energy into the tissue with low optical losses. However, their rigid and stiff nature might cause large tissue lesions, resulting in poor compatibilities with biological systems [28,29,30]. To resolve these challenges, implantable fibers and waveguides made from biocompatible and biodegradable materials have been actively explored.

In this article, we provide an overview on the recent advances of implantable optical fibers and waveguides for biomedical applications. In addition to inorganic fibers, progresses in flexible, stretchable, biocompatible and even biodegradable fibers and waveguides are discussed, associated with related materials, manufacturing strategies, device performance and functionalities. In particular, Table 1 summarizes representative materials and methods to form these fibers and waveguides, which will be explained in detail in Section 2. In Section 3, we highlight their applications in fields including optogenetics, phototherapy, sensing and imaging.

## 2. Materials and Synthesis

**Figure 1:**
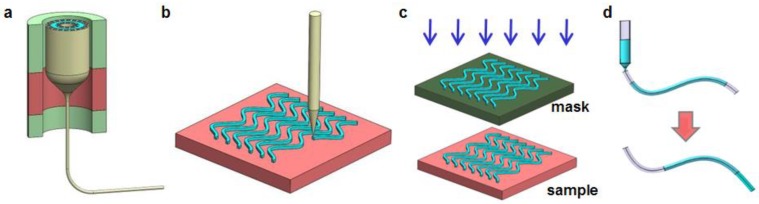
Schematic overview of representative approaches for fiber/waveguide fabrication. (**a**) Thermal drawing. (**b**) Printing. (**c**) Lithography. (**d**) Molding.

Figure 1 provides an overview of representative approaches to synthesize and fabricate implantable fiber or waveguides. Thermal drawing (Figure 1a) is commonly used for conventional optical fibers made of silica or other inorganic materials. Employed with custom-designed drawing towers [31,32], multifunctional polymer fibers with delicate structures can also be drawn from designed preforms [33]. Fibers fabricated with such drawing processes will be discussed in detail in Section 2.1 and Section 2.6. Three-dimensional (3D) printing (e.g., direct ink writing, Figure 1b) is a commonly used technique to develop implantable fibers and waveguides derived from organic materials. The inks flow through the printer nozzle at a controlled speed and then experience rapid solidification, forming optical waveguides and other photonic structures with desired shapes. As examples, Section 2.2 discusses biocompatible silk optical waveguides [34] developed by printing. Alternatively, lithographic techniques (Figure 1c) can be applied to fabricate waveguides, fluidic channels [35], etc. with features at micro- and even nanoscale. Molding (Figure 1d) is also an effective way to manufacturing optical fibers at low costs. In particular, Section 2.3 will discuss the development of hydrogel fibers [36], by ultraviolet (UV) curing optical materials in transparent tubes along with a dip-coating process to form claddings after extrusion. Molding techniques can also be applied to form fibers with other materials including synthetic polymers and elastomers, which will be respectively discussed in Section 2.4 and Section 2.5.

Typical characteristics of various optical fibers and waveguides include their structural (e.g., porosity, crystallinity), optical (e.g., refractive index, loss), mechanical (e.g., Young’s modulus, bending stiffness) and thermal properties (e.g., glass transition and melting temperatures), which play critical roles in their versatile applications for different fields. These properties can be evaluated by various approaches, for example, using electron microscopy and X-ray diffraction for structures, ellipsometry and spectroscopy for optics, dynamic mechanical analysis and thermal analysis. 

### 2.1. Inorganic Materials

**Figure 2:**
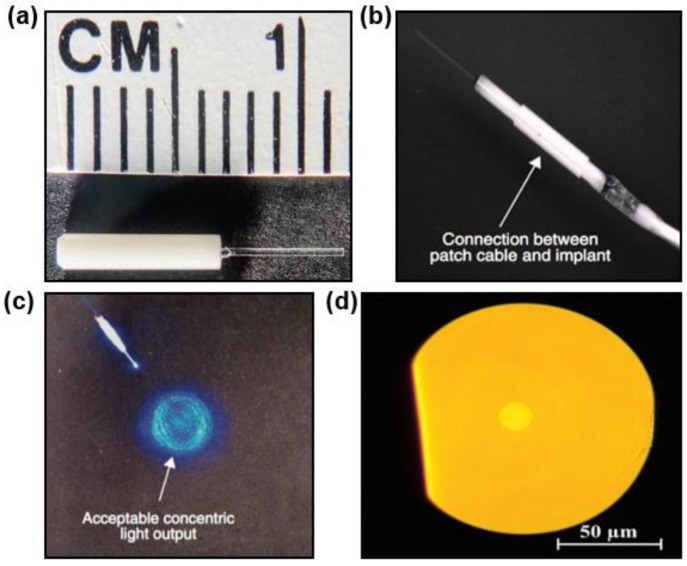
Inorganic fibers. (**a**) A silica fiber for tissue implant. (**b**) A silica fiber connected with a patch cable. Reproduced with permission [27]. Copyright 2011, Nature Publishing Group. (**c**) Transmission of light through a silica fiber. Reproduced with permission [27]. Copyright 2011, Nature Publishing Group. (**d**) The cross-sectional view of a calcium-phosphate glass based optical fiber. Reproduced with permission [38]. Copyright 2016, OSA.

Inorganic materials such as glasses (i.e., fused silica) are the base components for optical fibers (Figure 2a) and essential materials in the field of optics. Silica-based materials exhibit high optical transparency in a wide range of wavelengths from visible to near-infrared (near-IR), rendering optical fibers with extremely low propagation losses (as low as 0.2 dB/km around 1550 nm) [37]. Based on these glass materials, fibers with core-cladding structures can be formed at high temperatures using the thermal drawing method. The step or gradient refractive index profiles ensure the total internal reflection within the fibers and optical confinement in the core region.

Given that refractive indices of various biological tissues are generally ranged from 1.33 to 1.51 [11], the core-cladding structure confines photons into silica fibers with total internal reflection and guide light into deep tissues with negligible losses. The step-index structure has also been applied to the design of novel implantable optical fibers, which will be discussed hereinafter. Another advantage of silica-based implantable optical fibers lies in their chemical inertness, which ensures their long-term stability during operation. Standard silica optical fibers, inserted into a ceramic ferrule, can be connected with external light sources including LEDs and lasers by a cannula (Figure 2b), and serve as standard tools for optical neural interfaces [27]. As shown in Figure 2c, the output light profile from a silica fiber generally exhibits a uniform and centric power distribution.

Alternatively, highly transparent, degradable calcium-phosphate glasses (PGs) are considered as a novel category of materials for implantable photonic devices [38,39]. A new optical fiber made of this material recently has been manufactured using the thermal drawing technique [38]. Figure 2d presents a cross-section view of such a fiber. The fiber’s diameter is 120 μm and the core diameter is 12 μm. With different fractions of calcium oxide (CaO) and magnesium oxide (MgO), the refractive index of the PGs can be adjusted and then proper PGs are chosen to form a core-cladding structure with a step-index profile. Based on such structures, single-mode fibers can be formed and the measured optical loss of the fiber is as low as 1.86 dB/m at the wavelength of 1300 nm. The propagation loss at visible wavelengths is slightly higher and reaches 4.67 dB/m at 633 nm. The biodegradation of PGs fibers core is evaluated in physiological conditions. It demonstrates a decrease of fiber diameters along with the weight losses. In addition, the fiber degradation rate varies with PGs’ compositions, and higher CaO:MgO ratio leads to higher degradation rate.

Silicate or phosphate glasses provide ideal optical properties for low-loss fibers and waveguides. PGs based fibers also demonstrate their utility as fully bioresorbable implants. However, their rigidness and fragile characteristics render undesirable biocompatibilities with biological systems and then restrict their practical biomedical uses. Organic materials with better biocompatibilities have become promising candidates for optical implants, which will be discussed subsequently. 

### 2.2. Natural Materials

Naturally derived materials with ideal biocompatibility and biodegradability have been widely investigated for a variety of medical uses, such as drug delivery [40,41], tissue engineering [42,43], sensing and imaging [44,45]. To date, various optical waveguides have been fabricated using natural materials such as silk [34], cellulose [46] and bacteria cells [47]. For example, bio-derived cellulose polymers can be thermally drawn to form a core-cladding fiber structure, with some cellulose powders in between to form a hollow channel for potential drug delivery [46]. The propagation loss is measured to be ~1 dB/cm. These cellulose fibers are fully dissolved after 1-day immersion in aqueous solutions and during the dissolution, the light transmittance increases with water intake. Optical waveguides can also be formed from bacterial cells (e.g., Escherichia coli, propagation loss ~0.23 dB/μm [47]), and their optical losses are needed to be further reduced for practical uses.

In recent years, silk fibers produced by *Bombyx mori* worm or spiders have been studied and their application in the medical field are extensively explored [48,49]. To enable the silk’s capability in device applications, it is firstly converted to silk fibroin in a thin-film form through reverse engineering of the natural fiber generation process. The desirable biocompatibility and biodegradability of silk-based materials make them suitable for implantable devices that can be left in the body and are gradually resorbed by biological systems. Furthermore, silk materials exhibit mechanical properties similar to those of tissues and also offer distinctive optical properties. The combination of all these perfect properties makes silk an ideal material for a wide range of optical and photonic devices in medical applications [50]. 

**Figure 3:**
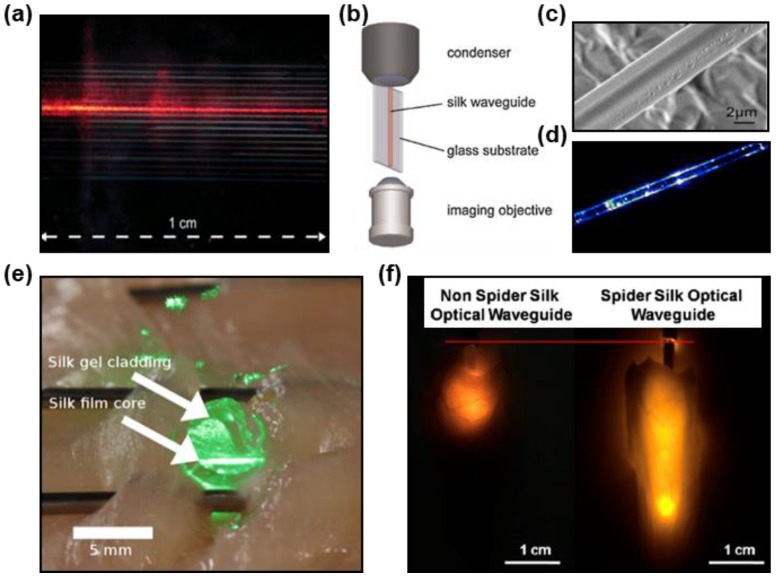
Waveguides made of natural materials. (**a**) Silk optical waveguide. Reproduced with permission [34]. Copyright 2010, Wiley-VCH. (**b**) Tools to analyze transverse faces of waveguides. Reproduced with permission [34]. Copyright 2010, Wiley-VCH. (**c**) Scanning electron microscopic (SEM) image of a silk fiber. Reproduced with permission. [54] Copyright 2013, AIP. (**d**) Micro-beam profile of a spider silk fiber. Reproduced with permission. [54] Copyright 2013, AIP. (**e**) An implanted silk optical fiber in tissue. Reproduced with permission. [51] Copyright 2015, OSA. (**f**) Comparison of light penetration in different waveguides. Reproduced with permission [55]. Copyright 2017, ACS.

Optical waveguides made from high-index (*n* = 1.54) regenerated *Bombyx mori* silk fibroin (Figure 3a) were fabricated on quartz (*n* = 1.52) via a printing based method [34]. The fabrication process of printed silk optical waveguides is schematically illustrated in Figure 1b. A position-adjustable condenser is applied to couple light inside the waveguide so that the transverse face is imaged and analyzed (Figure 3b). The average propagation loss of these waveguides is ~0.5 dB/cm. Recent investigations have demonstrated a biocompatible step-index optical waveguide made of silk fibroin [51,52]. This waveguide is fabricated with a high-index silk film (*n* = 1.54) for the core and a low-index silk fibroin hydrogel (*n* = 1.34) as the cladding layer, using injection-molding process. These silk waveguides are able to guide light through tissue with a propagation loss of ~2 dB/cm, which is mainly attributed to the rough edge of the silk film. An example of light guiding through chicken breast tissues via a silk optical waveguide coupled to a single mode glass optical fiber and a green laser source is shown in Figure 3e [51]. Silkworm gut fibers are fabricated by extracting and stretching the silk glands, which can serve as light-diffusion waveguides with a rough surface leading to massive light scattering and have been demonstrated for an optical stimulation on cell proliferation [53].

Unlike the natural and regenerative silkworm silk that have been widely studied through years for developing waveguides, less attention has been paid to the spider silk due to challenges of manufacturing at a large scale. Recently, a new class of optical fibers made from native spider silk (*n* = 1.50) through a molding process was presented [54]. These fibers have a diameter of ~5 μm, which are much thinner compared to conventional ones. The electron microscopy image shows a smooth surface of these fibers (Figure 3c). Figure 3d illustrates light guidance through the fiber, with a propagation loss of ~10.5 dB/cm. Native spider silk fibers are also able to deliver light in physiological liquid and in an integrated photonic chip. Through genetic engineering, producing spider silk proteins at a large scale becomes possible [55]. Recently, researchers have fabricated optical waveguides by exploiting genetically engineered spider silk proteins [55]. The refractive index (*n* = 1.70) of these fibers is much higher than that of biological tissues (*n* = 1.33–1.51). In addition, with a low propagation loss of ~0.8 dB/cm lower than that of regenerative silkworm silk waveguides, these waveguides are capable of guiding and delivering light and energy into deep tissues. As represented in Figure 3f, after inserting the recombinant spider silk optical waveguide, the penetration length of the light into muscle increases from less than 1 cm to 3 cm [55].

### 2.3. Hydrogels

Hydrogels can be easily integrated within biological systems, because of their mechanical properties similar to those of biological tissues, as well as their high water content and porous structures [56,57]. These attributes allow them to encapsulate living cells and make them ideal components of bio-scaffolds in tissue engineering [57]. Hydrogels with optimized chemical and physical compositions act as favorable materials for light guiding [2]. Based on their capability of cell encapsulation, various cell-based optical sensing and phototherapy can be carried out in vitro or in vivo [58]. In addition, hydrogel based optical waveguides have potentials in various applications including drug delivery [59], optogenetics [58] and so on.

As an example, hydrogel-based planar optical waveguides are manufactured out of agarose and gelatin via spin coating [60]. The core region is made by cross-linked gelatin with a refractive index of 1.536, while the cladding layer is made by agarose with a refractive index of 1.497. Both agarose and gelatin are bio-derived polymers that meet the need of biocompatibility and biodegradability. Via spin-coating or direct molding, the size of planar waveguides can vary from a few μm to mm, with the potential for implantable applications. Agarose-based hydrogels are also exploited to form an optical waveguide integrated with a fluidic channel by soft lithography [35]. Materials of two different gel concentrations with a refractive index difference are applied, forming a two-layer structure to guide light effectively. A microfluidic channel is built on top of the waveguide for on-chip biosensing applications.

**Figure 4:**
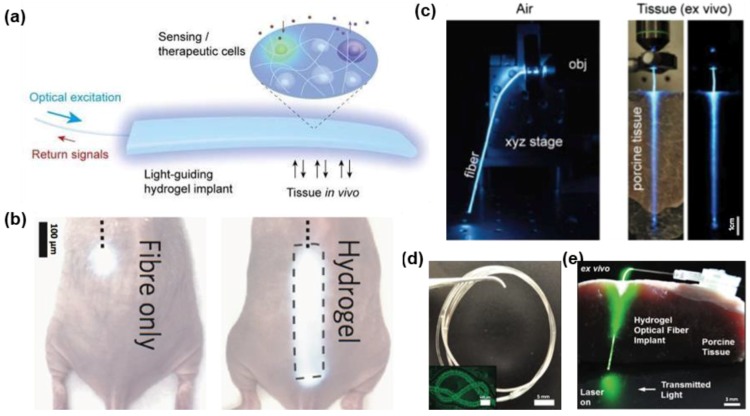
Hydrogel based waveguides. (**a**) Illustrating scheme of a light-guiding hydrogel with encapsulated cells. Reproduced with permission [58]. Copyright 2013, Nature Publishing Group. (**b**) Comparison of light scattering profiles with (**left**) and without (**right**) hydrogel implants. Reproduced with permission [58]. Copyright 2013, Nature Publishing Group. (**c**) Hydrogel fiber guiding light in air (**left**) and porcine slices (**right**). Reproduced with permission. [36] Copyright 2015, Wiley-VCH. (**d**) Image of a hydrogel optical fiber. Reproduced with permission [62]. Copyright 2017, Wiley-VCH. (**e**) Insertion of a fabricated hydrogel fiber in tissue. Reproduced with permission [62]. Copyright 2017, Wiley-VCH.

Shown in Figure 4a, hydrogels based on polyethylene glycol diacrylate (PEGDA) are utilized to fabricate slab waveguides using mold-injection and ultraviolet (UV) cross-linking processes, with particular applications in optogenetic stimulation and cell-based toxicity sensing [58]. Specifically, living cells can be encapsulated into the waveguide structure and perform sensing or therapeutic operations. In addition, excellent transparency allows PEGDA waveguides to simultaneously deliver excitation light and collect fluorescence signals by coupling to standard silica fibers. The optical properties of PEGDA hydrogels with different concentrations and molecular weights are characterized. Other properties, including stiffness, cell viability and swelling ratio, are taken into account. Hydrogels with a molecular weight of 5 kDa and a 10% *w*/*v* concentration are chosen to form implantable waveguides with an average optical loss of 0.23 dB/cm in the blue-green spectrum (from 450 nm to 550 nm). Figure 4b compares light guiding properties within the tissue with or without a hydrogel waveguide coupled to an external light source. By implanting the slab waveguide, the illumination area is expanded by 40 times.

To improve the light-guiding efficiency, step-index optical waveguides based on hydrogels are developed by molding and dip coating [36]. The difference of refractive indices between the polyethylene glycol (PEG) based core and the calcium alginate based cladding is critical for light confinement in waveguides. PEG has refractive index of about 1.46, while the low concentration of sodium alginate as the second hydrogel has a lower refractive index that is close to water (about 1.34). Figure 1d illustrates the molding process to form the core of such step-index hydrogel-based optical fibers. In the core, a platinum-cured silicone tube is used as a mold and filled with a hydrogel precursor solution, which is subsequently cross-linked through UV irradiation. Then the dip coating method is used to encapsulate the core with Ca^2+^ crosslinked hydrogels. In this process, the diameter of optical fibers could be adjusted by the number of dipping times. Figure 4c demonstrates the light guidance of such step-index optical fibers in the air and biological tissue, respectively. With a low optical loss (0.3 dB/cm in the air and 0.49 dB/cm in tissue) in visible ranges, these fibers have been implanted into live mice to justify the capability in sensing and photomedicine.

Fragile characteristic of hydrogel photonic devices is the result of weakness of common synthetic hydrogels which possess a brittle nature. These poor mechanical properties would cause difficulty for these hydrogels to be applied in implantable devices because during body movements, they might cause damage to the tissue. Therefore, improving the mechanical properties and stability of hydrogel fibers has become critically important [61], especially for biosensing applications. A novel hydrogel material with enhanced toughness and stretchability has been applied for optical fibers [61]. Specifically, a hybrid polymer network that contains both ionic and covalent bonds is introduced into hydrogels, improving the material robustness. Following this principle, highly stretchable hydrogel fibers are developed for strain sensing. Combining Ca^2+^ crosslinked alginate and covalently crosslinked polyacrylamide (PAAm), tough hydrogels are synthesized via UV polymerization (for the core) and dip coating (for the cladding). A 1.1 mm-diameter fiber can be stretched up to 730% and repeated 100 times (with 300% strain) without apparent plastic deformation. The optical absorption of dye-loaded fibers is proportional to the applied strain based on which the strain sensing function has been demonstrated. Such stretchable waveguides are envisioned to be used as optical strain sensors in wearable devices. In addition, another class of optical fibers has been recently developed for glucose sensing [62]. In this work, a hydrogel fiber is demonstrated, which is based on poly(acrylamide-co-poly(ethylene glycol) diacrylate) p(AM-co-PEGDA) for the core region and cladded with Ca^2+^ alginate and functionalized with phenylboronic acid. Figure 4d,e illustrate these hydrogels based optical fibers as well as their capability of guiding light through porcine tissues. Refractive indices of the cladding and the core are measured to be 1.34 and 1.46, respectively. The low optical loss measured in tissue phantoms is about 0.28 dB/cm. Their unique mechanical properties attract lots of attention in medical applications such as phototherapy and photomedicine [63]. Researchers have also manufactured a fluorescence slab waveguide made of hydrogels and doped with carbon dots to detect heavy metal ions, such as Hg^2+^ ions [64]. These waveguides exhibit smooth surface and obtain desirable transparency in the range of 400–800 nm, with an average propagation loss of less than 1.25 dB/cm.

### 2.4. Synthetic Polymers

**Figure 5:**
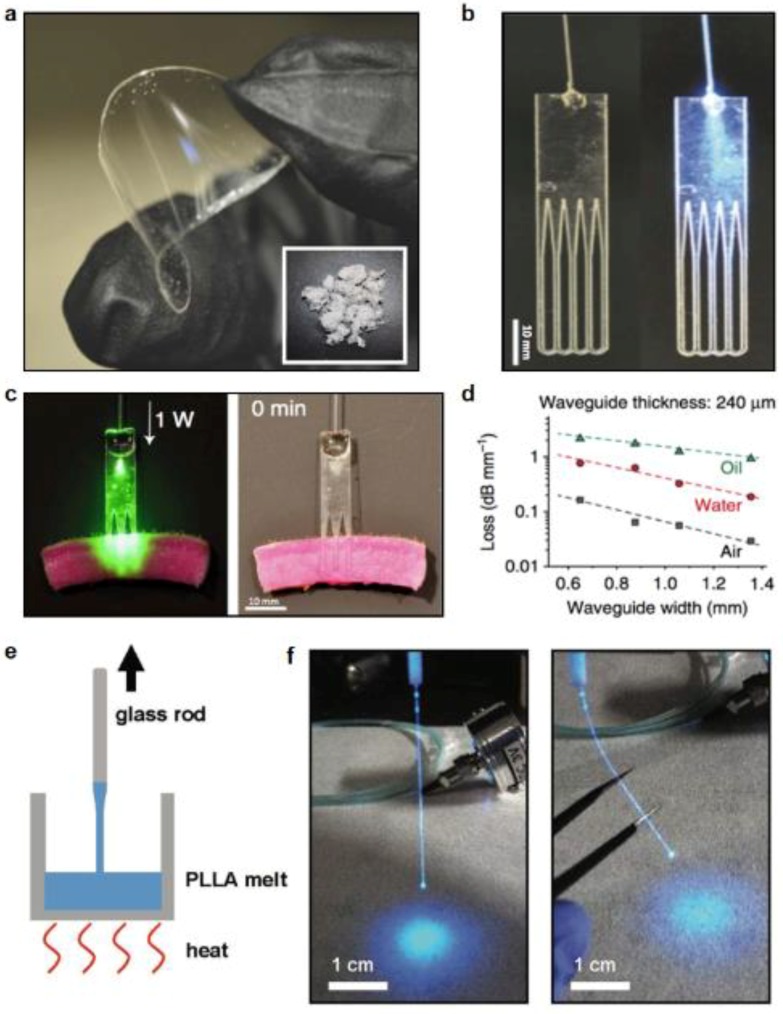
Fibers and waveguides made of biodegradable synthetic polymers. (**a**) A transparent, flexible PLLA film. Reproduced with permission [68]. Copyright 2016, Nature Publishing Group. (**b**) A comb-shaped PLLA waveguide. Reproduced with permission [68]. Copyright 2016, Nature Publishing Group. (**c**) Light delivery into deep tissues via the waveguide. Reproduced with permission [68]. Copyright 2016, Nature Publishing Group. (**d**) Measured optical loss of the waveguide in different media. Reproduced with permission [68]. Copyright 2016, Nature Publishing Group. (**e**) Process of forming thermally drawn PLLA fibers. Reproduced with permission [69]. Copyright 2018, Wiley-VCH. (**f**) A cylindrical PLLA fiber connected to a blue LED. Reproduced with permission [69]. Copyright 2018, Wiley-VCH.

In the past, biodegradable synthetic polymers like poly (lactic acid) (PLA), poly (lactic-co-glycolic acid) (PLGA), and polycaprolactone (PCL) have been extensively explored for use as structural materials for bio-interfaces [65,66]. Degradation rates of these synthetic polymers are controllable and vary with molecular weights, chemical compositions (e.g., lactide/glycolide ratio) and aqueous environments. They have been widely used as biomedical implants like suture, stents and various injectable products. Although these polymers are mostly utilized as implantable structural materials in opaque forms, amorphous states of these polymers are capable of guiding light in tissue because of their low extinction and high refractive indices (~1.46), which are well-suited for optical waveguide devices. PLA and PLGA based implantable waveguides are fabricated by melt pressing and laser cutting, as shown in Figure 5a,b [67,68]. These waveguides are coupled to external light sources to direct light into the deep biological tissue for treatment. Photochemical tissue bonding (PTB) treatment of a full-thickness skin incision is successfully demonstrated. The modulation of the waveguide surface pattern can help optimize the output light profile by inducing bending loss and an optimal waveguide is demonstrated with a uniform light distribution for photobleaching (Figure 5c). The propagation losses of these bioresorbable waveguides are measured in different environment and presented in Figure 5d.

Recently, poly(L-lactic acid) (PLLA) based optical fibers are fabricated via thermal drawing process [69]. Figure 5e schematically illustrates the fabrication process. In this method, PLLA powders are melted at 220 °C and fibers are formed through a crystalline-to-amorphous phase transition. Fiber diameters are manipulated by controlling the drawing speed. Figure 5f shows a formed PLLA fiber with a diameter of around 200 μm coupled to a blue LED light source. Due to the transparency of PLLA in the visible ranges, these optical fibers with cylindrical structures are able to deliver light in the air with a propagation loss of 1.64 dB/cm. The higher refractive index of PLLA (*n* = 1.47) than that of tissue makes PLLA fibers guide light into deep tissues effectively. These implantable fibers are applied as neural interfaces including optogenetics and fluorescence detection. With experiments in living mice, in vivo brain function modulations including neural sensing and interrogation are realized, along with a gradual biodegradation.

### 2.5. Elastomers

Optical waveguides made of polydimethylsiloxane (PDMS) based elastomers (*n* = 1.42) are demonstrated via molding process. The specific feature of this waveguide is the ability to carry adequate amounts of light uniformly by a tapered structure, contributing to the scleral cross linking treatment [70]. Recently, a fully biodegradable step-index optical fiber made from elastomers are developed [71]. For this biodegradable fiber, the core is made of poly(octamethylene maleate citrate) (POMC) while the cladding layer is formed with poly(octamethylene citrate) (POC). Citrate-based elastomers have been studied as biodegradable implants for tissue engineering, drug delivery, etc. and the monomer, citric acid, is an intermediate of metabolism. The elastomer fibers exhibit high flexibility (initial modulus ~3.39 MPa) and stretchability (elongation ~61.49%). With a measured propagation loss of ~0.4 dB/cm, they are capable of carrying sufficient amount of light deep to tissue. The degradation time (ranging from few weeks to years) of these fibers depends on the synthesis and structure of citrate-based elastomers. These developed step-index fibers are applied for in vivo fluorescence detection to study deep inside tissue. Besides, the image transmission function has also been displayed.

### 2.6. Hybrid Materials

Biomedical implants with versatile functions can help to realize a multimodal sensing and (or) stimulations, for instance, a real-time interrogation and monitoring of neural circuits using optical, electrical and chemical signals [33,72]. Such multifunctional waveguides can be fabricated from hybrid materials by integrating conductive wires (or coatings) and hollow channels with optical fibers. Most of the devices that had been used for recording the neural activities were mostly fabricated out of metals, semiconductors or glass which can easily cause harm to the surrounding tissues during animal body’s movement because of their rigidity or stiffness characteristics. Polymer materials are employed for multifunctional fibers, considering their flexibility and excellent transparency which makes them suitable for the purpose of implantation as neural interfaces. To develop the delicate structure of these multifunctional fibers, thermal drawing process is used based on a well-designed preform as a starting structure so that different functions can be integrated in one fiber probe [31]. These hybrid materials should have similar mechanical and thermal properties to form the deterministic fiber structures. Moreover, in order to guide light, the internal and external layers of such optical fibers are required to have different refractive indices. Conductive polymers or silver nanowire coatings are integrated to record the electrophysiological signals. Meanwhile, hollow channels are developed for drug delivery and other fluidic injection. By controlling drawing temperatures and speeds, researchers can achieve the geometry of the device to fit specific applications.

**Figure 6:**
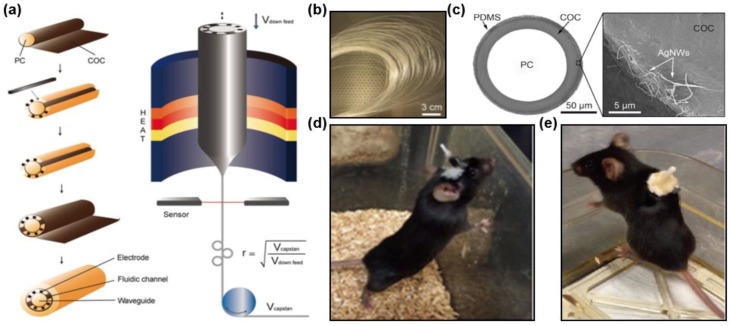
Hybrid, multifunctional fibers. (**a**) Steps involved in the fabrication of multifunctional fiber device. Reproduced with permission [72]. Copyright 2017, Nature Publishing Group. (**b**) Multifunctional fiber spools. Reproduced with permission [74]. Copyright 2017. AAAS. (**c**) Cross-sectional view of the fiber (right panel) and SEM image of the AgNW electrodes (left panel). Reproduced with permission [74]. Copyright 2017, AAAS. (**d**,**e**) Living mice with the implanted fiber probes in the brain and spiral cord, respectively. Reproduced with permission [72,74]. Copyright 2017, Nature Publishing Group and AAAS.

On the basis of this idea, following materials are selected to fabricate hybrid-materials fibers for the use of optical neuromodulation: polycarbonate (PC; refractive index *n* = 1.58; glass transition temperature *T_g_* = 145 °C; Young’s modulus *E* = 2.38 GPa) and cyclic olefin copolymer (COC; *n* = 1.52, *T_g_* = 158 °C, *E* = 3.0 GPa) [33,73]. Figure 6 schematically illustrates the thermal drawing method and steps of the fabrication to produce a multifunctional fiber. The structure of the fiber consists of an optical waveguide made of PC as the core and COC for the cladding, six electrodes made of conductive polyethylene with 5% graphite (gCPE), two hollow microfluidic channels and another PC based cladding layer for protection. The propagation loss is measured to be 1.6–2.6 dB/cm. The impedance at 1 kHz of the fiber electrode is approximately 1 MΩ under bending or flat conditions. The device can be implanted into animal brains and operated for a long time (about two months) considering their flexibility and small dimensions (Figure 6d). The fiber is able to simultaneously deliver drugs into the neuron cells, stimulate optical signals and record electrophysiological activities in one step. Specifically, viral injection is performed through the hollow channel of the multifunctional fibers and then colocalized protein expression, illumination and recording can be achieved in living animals [72]. 

With similar fabrication process, another kind of hybrid fibers is developed with stretchable materials, aiming to probe the spinal cord circuits of freely moving mice (Figure 6b). Similar materials as fibers shown in Figure 6a are selected for the core and the cladding, combining with polydimethylsiloxane (PDMS). A mesh of transparent silver nanowires (AgNWs) with a wire diameter of 70 nm and a length of 40 mm is dip-coated alongside the fiber and encapsulated with PDMS (Figure 6c), producing a conductive layer to collect electrophysiological signals. The electrode impedance is around 50 kΩ and can be tuned by choosing different concentrations of AgNWs solutions. Full elastomer-based AgNWs-coated fibers are formed based on COC elastomer as a core and PDMS as a cladding. The fiber can be stretched to 100% strain by integration 3 layers of AgNWs while maintain its electrical conductivity. The stretchable fibers are implanted into the lumbar region of the spinal cord (Figure 6e) and both spontaneous and optical stimulated neural signals can be recorded [74].

## 3. Biomedical Applications

**Figure 7:**
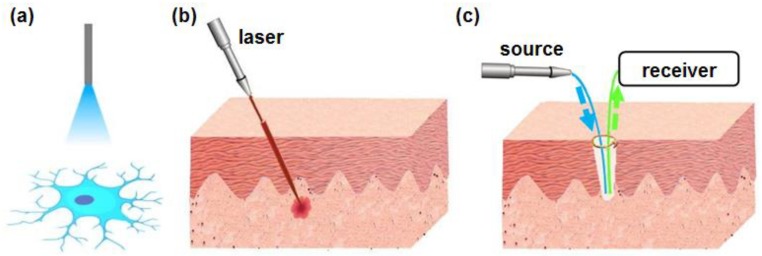
Schematically illustrated examples of applications for implantable fiber/waveguides. (**a**) Optogenetics. (**b**) Laser surgery. (**c**) Fluorescence sensing.

In the previous section, we discussed the materials and fabrication processes for biocompatible and implantable optical fibers and waveguides. Fibers and other photonic structures based on biocompatible materials such as ceramics, hydrogels, synthetic polymers and even natural materials have been exploited in biomedical applications ranging from optogenetic stimulation, fluorescence photometry, surgery, phototherapy, to biochemical sensing and imaging. In this section, we provide examples of their wide applications in the development of optogenetics, surgery and phototherapy, optical sensing and bio-imaging. The cartoons in Figure 7 demonstrate three representative biomedical applications: optogenetics (Figure 7a), laser surgery (Figure 7b) and fluorescence sensing (Figure 7c), in which implantable fibers or waveguides play an important role.

### 3.1. Optogenetics

**Figure 8:**
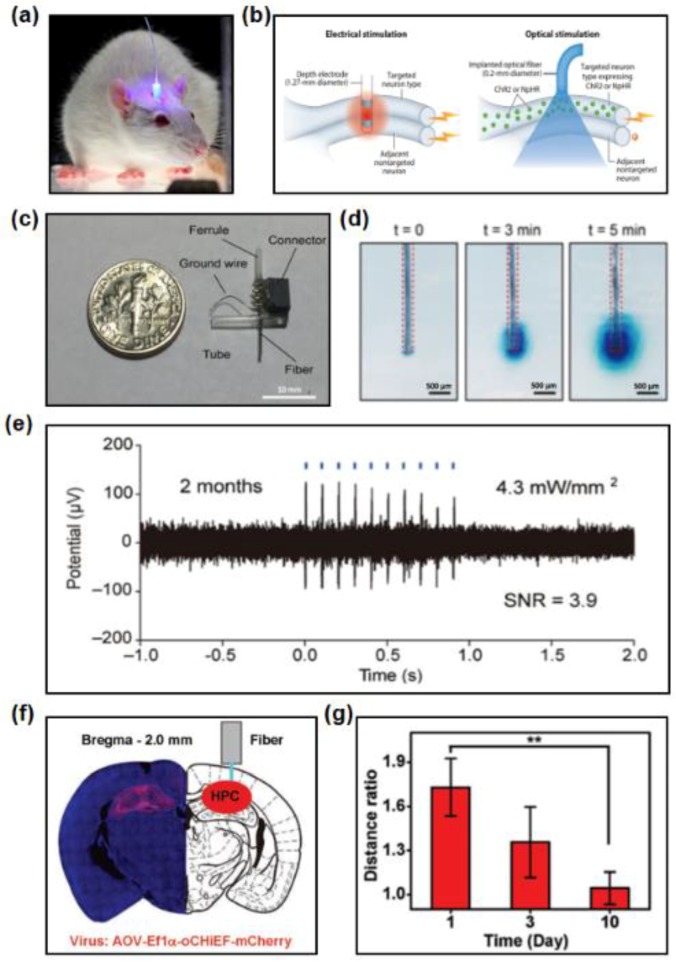
Application in optogenetics. (**a**) A fiber implanted into a freely behaving mouse. Reproduced with permission [76]. Copyright 2015. (**b**) Comparison of electrical simulation (**left**) and optical stimulation (**right**) for neural cells. Reproduced with permission [20]. Copyright 2014, Annual Reviews. (**c**) A fiber probe equipped by an optical ferrule, an electrical connector, and an injection tube. Reproduced with permission [72]. Copyright 2017, Nature Publishing Group. (**d**) A multifunctional fiber with integrated microfluidic channels for fluidic injection into the brain. Reproduced with permission [72]. Copyright 2017, Nature Publishing Group. (**e**) Electrophysiological traces under optical stimulation. Reproduced with permission [72]. Copyright 2017, Nature Publishing Group. (**f**) Confocal microscopic image of a coronal section after full expression (**left**) and schematic diagram of fiber implanted into coronal (**right**). Reproduced with permission [69]. Copyright 2018, Wiley-VCH. (**g**) Ratio of measured distance of mice travelling with and without optical stimulation. Reproduced with permission [69]. Copyright 2018, Wiley-VCH.

Optogenetics, the combination of optical and genetic methods to activate or deactivate certain events in specific cells, is employed today to study activities of neurons and their related behavior. Over the decades, it has enabled acquisition of invaluable insight into a wide range of fields in physiology, pathology, behavior and even psychiatry. Optical fibers and waveguides, along with microbial opsins and vectors, are most commonly core features in optogenetics. Specific opsin gene expression, carried to well-defined cells by viral vector, encodes a protein that causes electrical current across cell membranes when illuminated by light. With excitation light delivered by optical fibers and waveguides, targeted cells exhibit events of interest. Optogenetic methods have become standard tools for studying neural circuits underpinning behavior in freely behaving animals (Figure 8a). Here, we provide studies exploring implantable, biocompatible optical fibers and waveguides with various functions for optogenetics [75].

Optical stimulation is often compared with electrical one in causing or inhibiting activities of neurons in brain regions. Although electrical stimulation has been employed in both experimental and clinical level to probe and control neural activities in discrete brain areas, it fails to control cells specifically, as shown in Figure 8b [20]. Optical stimulation, by contrast, targets only neuron type expressing microbial opsins (ChR2, NpHR, etc.). This characteristic also sheds light on single-cell control and cellular signal traveling.

Recently, much attention has been drawn to the development of optical fibers that can realize several functions with one fiber. Stretchable probes are demonstrated, which are made of polymer fiber coated with conductive meshes of silver nanowires [74]. These probes have the ability to simultaneously stimulate neurons and record electrophysiological activity. With flexible and stretchable characteristics, such fibers can be tailored for stimulating and monitoring electrophysiological activities in spinal cord. Park et al. made further attempt by integrating all steps of optogenetics in a single biocompatible platform [72]. These fibers contain micro channels, electrodes and waveguides core to enable viral vector injection, optical stimulation and simultaneous electrophysiological recording, all within the general dimensions of fibers used in optogenetic studies. Figure 8c shows the probe equipped by an optical ferrule, electrical connector, and an injection tube to fit its functions. Microfluidic channels within fibers deliver liquid into deep brain tissue efficiently during the tests in Figure 8d. The device maintains reliable optical stimulation capabilities for months, with high signal to noise ratio (Figure 8e). Such technology allows for one-step surgery, providing minimally invasive alternatives to the current practice in optogenetics.

Fully biodegradable and bioresorbable photonic systems are also the pursuit of continued research in optogenetics. PLLA based optical fibers are explored as tools for light delivery and detection, sparing the secondary damage during the retraction [69]. The virus specifically targeting hyperactivating hippocampus (HPC) neurons is injected into the bilateral HPC and the encoded protein expresses after two weeks. Then PLLA fibers coupled with a 473 nm laser source are implanted into the section (Figure 8f). When the light is guided to HPC via PLLA fibers, a seizure is induced, which activates the mice and results in increased travelling distance. Figure 8g illustrates ratio of travelling distance of mice with and without optical stimulation. The significant decrease of distance ratio indicates performance degradation of PLLA fibers within 10–15 days. These results suggest promising tools for fundamental biomedical research and even clinical uses.

In addition, there are some other designs for specific applications. Pisanello et al. demonstrated that optical fibers with small taper angles can be used to illuminate focal or broad brain volumes [77]. They also use focus ion beam to open multiple light windows on a tapered optical fiber, which allows simultaneous, selective stimulation of different brain regions and reduces the invasiveness of optogenetic device [78]. Optogenetics also shows potential in in vivo optical-sensing and light-controlled therapy. Choi et al. reported hydrogel waveguides encapsulating optogenetic cells for sensing of cytotoxicity and therapy in diabetic animals [58]. These works offer promising complement for existing methods in certain scenarios.

### 3.2. Phototherapy and Laser Surgery

With numerous lasers and optical devices applied in clinical practice to assist operation and cure diseases, light has exerted increasingly significant influence on medicine. Light-activated therapies, laser surgery, optical diagnostics and other emerging technologies are widely used in treating tumors, dermatosis, and so on, and sophisticated technologies have been developed to ease pains in patients and treat diseased tissue with accuracy [79,80].

**Figure 9:**
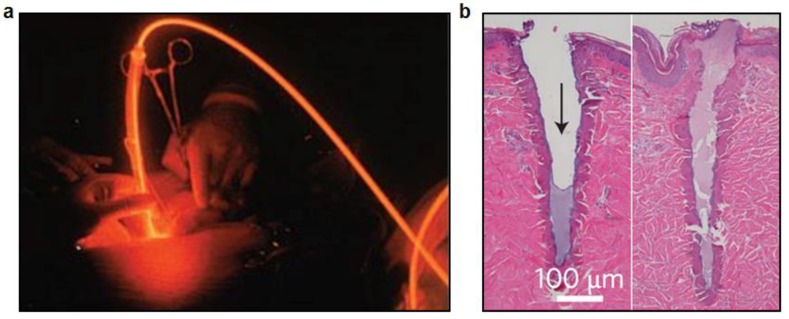
Applications in phototherapy and surgery. (**a**) An example of photodynamic therapy. Reproduced with permission [81]. National Cancer Institute. (**b**) An example of application of light in surgery. Reproduced with permission [1]. Copyright 2017, Nature Publishing Group.

Phototherapies with selected light wavelengths have become commonplace in clinic and are used in numerable cases. Furthermore, more efficient capabilities can be achieved by exogenous photosensitizers than intrinsic phototherapy, such as oxidation in photodynamic therapy [14]. With appropriated design strategies, photodynamic therapy has the ability to target diseased cells without damage to healthy cells. Today, photodynamic therapy is an established treatment now clinically employed to treat various cancers (Figure 9a). In clinical practice, its efficiency depends on many factors such as the total light exposure dose and light fluency rate [4], rendering convenient light delivery system as one of the future research directions. Therefore, implantable and biodegradable waveguides may effectively extend the therapeutic depth and time frame in photodynamic therapies, enabling chronic deep-tissue treatment in the future [68].

Other light-based technologies, such as flexible waveguides for periscleral cross-linking [70] and photochemical tissue bonding for full-thickness skin incision [68], provide new opportunities for further innovation in photomedicine.

Lasers, with extremely high emission intensities, can be generated in short pulses and selective wavelength and cause hazards to body tissues, a characteristic appreciated in surgery. Laser surgery has been routinely used in various medical fields including ophthalmology, dermatology, and tissue ablation, which is accomplished through fiber-optic delivery [1]. Photothermal induced damage can be filled with plug or clot within minutes, where new tissue will replace unwanted tissue (Figure 9b). Implantable fibers and waveguides possess the advantage of delivering light into deep tissue, and their applications in photothermal or photodynamic therapies show potential for deep-tissue light-induced therapies [68].

### 3.3. Optical Sensing

Here, we provide an overview on implantable fibers and waveguides with a focus mainly on their applications in sensing. To research on metabolism of living cells and their activities, bio-sensing is a good way to get the information. However, deep-tissue and real-time sensing is still a challenge, blocking the rapid disease diagnosis and researches in physiology, pathology, etc. Light detecting, which uses light to interact with biological systems, has the advantages of minimal invasiveness and high spatiotemporal resolution. Optical sensing offers advantages over its electrochemical counterpart since they can be label-free, conduct real-time continuous monitoring for long periods of time, and cause minimal damage to the body.

**Figure 10:**
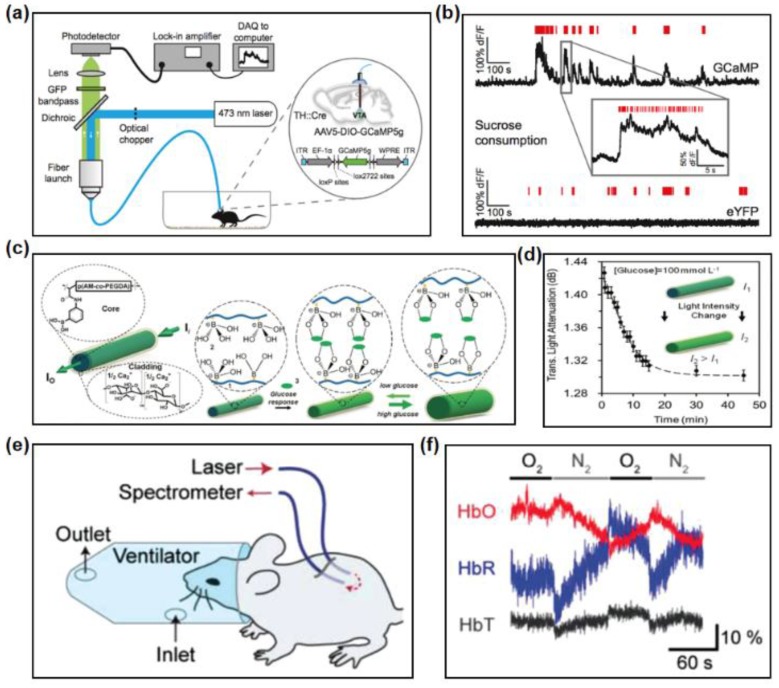
Fibers and waveguides in optical-sensing. (**a**) A scheme diagram of a fiber photometry system. Reproduced with permission [23]. Copyright 2014, Elsevier. (**b**) Recorded signals from the VTA of mice expressing GCaMP (**top**) and eYFP (**bottom**). Reproduced with permission [23]. Copyright 2014, Elsevier. (**c**) Scheme of the glucose-sensitive optical fiber. Reproduced with permission [62]. Copyright 2017, Wiley-VCH. (**d**) Attenuation of the transmitted light through the glucose-sensitive optical fiber. Reproduced with permission [62]. Copyright 2017, Wiley-VCH. (**e**) Reflectance oximetry of biological tissues. Reproduced with permission [36]. Copyright 2015, Wiley-VCH (**f**) Variation of typical time-lapse of calculated concentrations of oxy-hemoglobin, deoxy-hemoglobin and total hemoglobin. Reproduced with permission [36]. Copyright 2015, Wiley-VCH.

Figure 10a illustrates a fiber photometry system used to record neural projection activity underlying certain behavior [23]. An implanted fiber simultaneously delivers 475 nm excitation light and collects fluorescence emission from excited neurons in targeted region. After a dichroic mirror and optical filters, green fluorescence reaches the photodetector. Fluorescence intensity positively correlates with sucrose licking epochs (Figure 10b), indicating the relation between certain activities and targeted brain region. Deep brain fluorescence sensing can also be coupled with optogenetic interrogation to facilitate in vivo neuro activity study [69].

Optical fibers are investigated for application in real-time continuous glucose sensing. Yetisen et al. demonstrated hydrogel fibers consisting of poly(acrylamide-co-poly(ethylene glycol) diacrylate) cores functionalized with phenylboronic acid and cladded with Ca alginate [62]. Phenylboronic acid, a glucose-sensitive chelating agent, is incorporated into the core for sensing glucose. At different glucose concentrations, the chelation of glucose enables reversible changes of fiber diameter, and in response changes of refractive index of the hydrogel fiber. The intensity of light transmitted across the hydrogel fiber is the function of binding time, and gradually reaches the equilibrium (Figure 10d).

The application of optical fibers for blood oxygenation sensing has also been explored. Figure 10e shows a schematic illustration of reflectance oximetry of tissues using fibers [36]. One fiber delivers excitation light at 560 nm and 640 nm into the tissue, while the other collects the light through tissue, which is received by spectrometer. The blood oxygenation is regulated by oxygen level of supplied gas, which is switched between nitrogen and oxygen. The changes of optical intensity at these wavelengths are measured and converted to relative oxy- and deoxy-hemoglobin concentrations. The inhalation of nitrogen results in sudden decrease of oxy-hemoglobin and increase in deoxy-hemoglobin, representing the drop of blood oxygen concentration (Figure 10f). Such findings provide potential insight into the use of photonic tools toward sensing and monitoring in vivo system, pushing forward the field of photomedicine.

### 3.4. Optical Imaging

**Figure 11:**
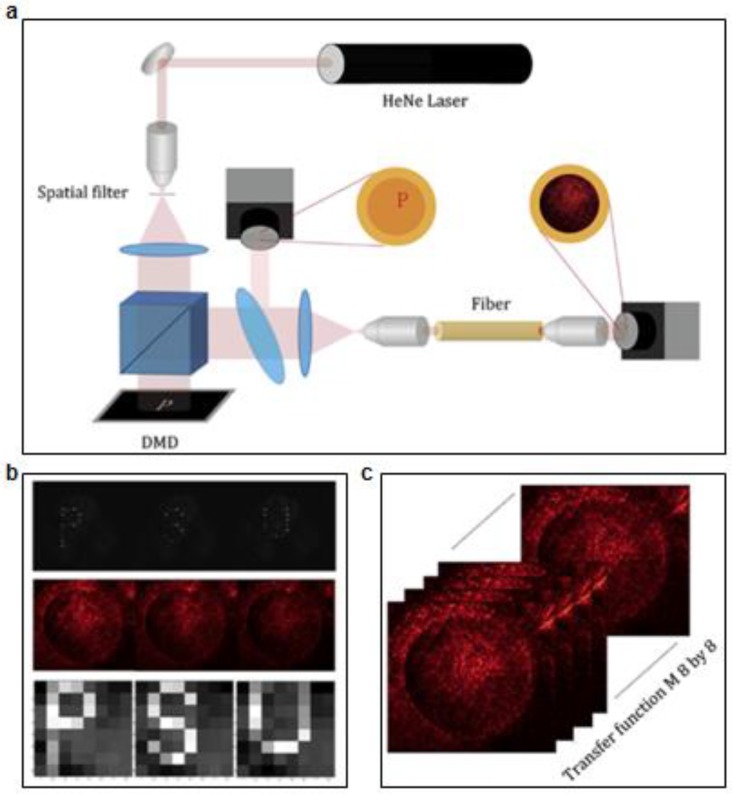
Bioimaging using a degradable fiber. (**a**) Experimental setup for the fiber imaging. Reproduced with permission [71]. Copyright 2017, Elsevier. (**b**) Initial projected letters (upper), the corresponding light output with speckle patterns (middle), and the reconstructed image (lower). Reproduced with permission [71]. Copyright 2017, Elsevier. (**c**) Collective images showing prerecorded impulse responses of the optical fiber. Reproduced with permission [71]. Copyright 2017, Elsevier.

From biological to medical fields, bio-imaging is a valuable tool to analyze the characteristics and conditions of cells or specific regions, and even expression and distribution of molecules. Optical imaging, with the characteristics such as high sensitivity, high resolution and high speed, has great potential in exploring mechanism of diseases, obtaining physiological information and diagnosing disease. Over the past decades, the development of optical imaging has deepened our understanding of the structure and physiological activities of living organisms. Early practice is in vitro imaging on biological section or cell culture, which is accurate but fails to get information directly from living tissue. The second method is by opening an optical window in anesthetized animals. Such practice provides high-quality imaging, but cannot support long-time monitoring and in moving animal, presenting a significant limitation for optical imaging. Implantable optical fibers and waveguides in tissues for light delivery and collection become efficient ways to alleviate these problems. Figure 11 shows an example of flexible biodegradable fibers for deep-tissue optical imaging, indicating the potential of image delivery function [71].

To meet the diversified requirement on optical, mechanical and biological functions, researchers present citrate-based polymeric optical fibers with POC cladding and POMC core. Figure 11a depicts the experimental setup of optical imaging. Spatial patterns on a digital micromirror device (DMD) are projected onto the proximal end of the fiber by laser. At the input end of the fiber, a beam splitter is applied to confirm the pattern projection, which transmits across the fiber and is recorded by a charge-coupled device (CCD) camera. Due to the multi-modal propagation, the output projection does not resemble to the input pattern. Figure 11b shows the initial projected letters, the corresponding random speckle patterned output and the reconstructed image. The pre-recorded impulse responses of the multi-mode optical fiber used for image reconstruction are illustrated in Figure 11c. Based on the calibrated impulse responses, the retrieved image through multi-mode fiber still has good resolution. These findings provide a potential implantable imaging platform for advanced tissue imaging, monitoring and so on.

## 4. Summary and Outlook

We have reviewed biocompatible and implantable optical fibers and waveguides made from inorganic materials, bio-derived natural materials, hydrogels, synthetic polymers, elastomers as well as hybrid materials. Their novel applications in biomedical fields, including optogenetics, laser surgery and phototherapy, biosensing and imaging, have also been summarized. The limited penetration depth of visible light to biological tissues has urged researchers to develop various tools to guide photons into targeted areas. Compared with implantable light sources and bioluminescence devices [2], optical fibers and waveguides offer a simple but effective approach to overcome the obstacle and also exhibit versatile functions by introducing novel materials, designing delicate structures and/or integrating other functional devices. 

Although silica fiber optics have been serving as the most widely used light-guiding device due to their minimal optical loss and stability, it needs to be taken into account for more clinical practice that the intrinsic stiffness and brittleness as inorganic materials result in inferior biocompatibility. Therefore, several kinds of soft materials are exploited to fabricate optical fibers and waveguides with ideal flexibility, stretchability and favorable biocompatibility. Similar to silica fibers, the thermal drawing method is adopted to fabricate polymer fibers. Multifunctional fibers are drawn from the well-designed preform so that multiple materials and novel structures can be integrated, realizing simultaneous optical interrogation, electrical recording and chemical delivery [33,72]. Besides the conventional thermal drawing method, molding, printing and microfabricating process are all employed to form a fiber or create a novel waveguide structure. Molding provides a universal solution to implantable fibers and waveguides, for example, combined with dip coating, step-index fibers are made from hydrogels or elastomers and demonstrate diverse functions for phototherapy, biosensing and imaging [36,63,71]. Printing and microfabrication method are mainly used for planar waveguide devices, among which the utility still needs further development. Another significant trend is the emerging biodegradable optical fibers and waveguides which can gradually disappear in vivo and need no retraction from body. Based on natural materials (silk, cellulose, etc.) which possess superior biocompatibility, researchers have manufactured biodegradable fibers and waveguides [46,52]. Biodegradable synthetic polymers (PLGA, PLLA, etc.) demonstrate advantages including ideal optical transparency, tunable degradation time and ease of processing. The PLLA fibers are applied as optical neural interfaces and the optical performance has been systematically studied during biodegradation [69].

The rapidly expanding biomedical applications are calling for both general and specialized studies on implantable fibers and waveguides. First, further decreasing the propagation loss is still highly demanding. Compared to silica optical fibers with a loss coefficient of a few dB/km, most of the newly developed fibers and waveguides have much higher losses (~dB/cm). The propagation loss includes intrinsic optical loss and scattering loss. Although it is difficult to eliminate the intrinsic loss, improvement of materials synthesis and processing methods can lower the light scattering resulted from impurities, rough surfaces, or interfaces. For example, core-cladding fibers with a chemical bonding cladding layer demonstrate a better optical performance [61]. Second, specialized functionalization with materials or design is definitely worth to enable further exploration for biomedical applications. For example, different phototherapy treatments require customized materials and devices to achieve efficacy considering the mechanical properties, robustness, light profiles and so on. In particular applications, light extraction behaviors are of critical importance and should be taken into account [68,70]. With implantable optical fibers and waveguides, various in vitro and in vivo optical sensors can be realized with capabilities of measuring optical intensity, wavelength shift, fluorescence, etc. Materials with novel functions, like phenylboronic acid for glucose sensing [62] and carbon dots for toxicity tests [64], need more explorations in future. Integrating with other implantable devices (photodetectors, optical filters, etc.), the sensing strategies of fibers/waveguides can be further expanded. Third, optimizing biodegradable fibers and waveguides is of great significance for clinical usage. Although different biodegradable materials have been exploited for optical fibers and waveguides, there are still a few challenges for them. Because of biodegradation, the optical performance decreases with time after implantation [69,71]. In the future, triggered materials can be applied to fabricate core-cladding fibers with improved stability and quick degradation after use. By cooperating with transient electronics [82], multiple functions can be achieved as fully biodegradable photonic devices and systems. 

It is envisioned that implantable fibers and waveguides will be built with better optical performance, flexibility and biocompatibility, and enhanced functionality. They will not only guide light to deep tissue, but also contribute to diagnosis, therapy and surgery in clinic. As a building block, they will also be an important part of complicated implantable photonic or optoelectronic devices, allowing diverse medical applications in future world.

## Figures and Tables

**Table 1:** A summary of representative materials and methods to form biocompatible fibers and waveguides.

Category	Materials	Fabrication Process
Inorganic materials	Silica, phosphate, silicon oxynitride	Thermal drawing, Lithography
Natural materials	Silk, cellulose, bacterial cells	Thermal drawing, Printing, Molding
Hydrogel	Agarose gel, PEG, alginate	Molding
Synthetic polymers	PLLA, PLA, PLGA	Thermal drawing, Molding
Elastomers	PDMS, POC-POMC	Molding
Multifunctional	COC, PC, CPE	Thermal drawing
